# *BJS Open* 2025 best colorectal surgery articles: editors’ choices

**DOI:** 10.1093/bjsopen/zraf181

**Published:** 2026-01-28

The *BJS Open* editors’ choices for 2025 recognize research that is, not only scientifically rigorous, but also immediately relevant to clinical practice. This year’s selection exemplifies how high-quality data can directly inform decision-making in modern colorectal surgery.

In a large cohort study of 490 patients with ulcerative colitis treated at one of Europe’s leading referral centres, the authors documented how medical and surgical management evolved over time^1^. These changes included the adoption of preoperative advanced therapies, delayed ileal pouch–anal anastomosis, and increased use of minimally invasive surgery. Despite these improvements, the incidence of pouch failure remained stable (overall rate 8.2%), with anastomotic leakage identified as the only significant risk factor.

A further collaborative study evaluated 401 patients undergoing stoma formation for Crohn’s disease across 44 centres^2^. Temporary stomas were reversed in 30.2% of patients at 6 months and 56.9% at 18 months, with an overall reversal rate of 63.3%. Ongoing medical therapy, patient fitness, and patient preference contributed to delayed or non-reversal. These findings highlight the risk of prolonged, and in some cases permanent, stoma-related morbidity.

In the field of oncology, a Swedish national prospective study assessed short-term outcomes of a watch-and-wait strategy in patients with rectal cancer achieving a clinical complete response after neoadjuvant therapy^3^. Between 2017 and 2023, 211 patients were included. At 6 months, 15.6% developed suspected local regrowth, with 32 of 33 undergoing abdominal resection. Salvage surgery was performed with curative intent in 94% of cases, while distant metastases occurred in 1.4%. These results align with existing literature and reinforce the oncological safety of early salvage surgery in carefully selected patients.

The colorectal community is increasingly aware of the worrying rise in early-onset colorectal cancer. The GEOCODE-Europe study analysed 851 patients aged 18–49 years across six European countries^4^. Most patients were diagnosed at age ≥39 years, 42% were overweight or obese, and 62.5% presented with stage III–IV disease. Tumours were predominantly distal. Significant geographical variation was observed in age at diagnosis, body mass index, disease stage, polyp prevalence, and family history. Mediterranean countries showed later age at diagnosis and more advanced disease, while non-Mediterranean countries demonstrated stronger familial associations. Further research is required to elucidate the aetiology underlying these trends.

Finally, an innovative study explored the potential role of artificial intelligence in clinical decision-making^5^. This retrospective single-centre analysis compared multidisciplinary team recommendations with those generated by ChatGPT-4 in 100 patients with newly diagnosed colorectal cancer. Pre-treatment concordance was observed in 72.5% of cases, increasing to 82.8% post-treatment. Discordance was more frequent in older patients and those with higher American Society of Anesthesiologists (ASA) grades. These findings suggest that large language models may have a future role as adjunctive decision-support tools.

We would like to thank all authors and peer reviewers for their continued support of the journal, whose commitment underpins the quality and integrity of *BJS Open*. We look forward to another successful year of innovation and collaboration in academic colorectal surgery in 2026.
